# Morbidity Profile of Patients With Sexually Transmitted Infections (STIs) and Their Determinants in a Tertiary Care Institute of Eastern India

**DOI:** 10.7759/cureus.78963

**Published:** 2025-02-13

**Authors:** Rajesh R, Koushik Shome, Prodip Sarkar, Souvik Manna

**Affiliations:** 1 Dermatology, Employee's State Insurance Corporation (ESIC) Medical College & Hospital, Alwar, IND; 2 Dermatology, Burdwan Medical College, Burdwan, IND; 3 Community Medicine, Employee's State Insurance Corporation (ESIC) Medical College & Hospital, Alwar, IND

**Keywords:** genital discharge, genital ulcer, sexually transmitted diseases, sexually transmitted infections, venereal diseases

## Abstract

Background: Sexually transmitted infections (STIs) have a tremendous health burden in India, owing to its younger population. The current study was conducted to study the pattern of different STIs and their determinants among cases presenting to a tertiary hospital STI clinic.

Methods: In this descriptive cross-sectional study, a total of 340 new cases of STIs attending the STI clinic during one year were enrolled. A semi-structured questionnaire was used to record the socio-demographic profile, sexual history, knowledge of STIs, and safe sex measures. A dermatologist performed the clinical examination, including a general and systemic examination.

Results: The mean age of the participants was 27.10 years (± 6.03) with a range from 17 to 45 years. The mean age at the first sexual encounter was significantly lower in females as compared to males (18.5 years versus 21.2 years, t=13.85, p=0.000). The most common morbidity among females was bacterial vaginosis (BV) (72, 43.4%), and the common STIs were herpes genitalis (52, 15.3%), *Candida *infection (49, 14.4%), non-gonococcal urethritis/cervicitis (43, 12.6%), trichomoniasis (42, 12.4%), genital warts (31, 9.1%), gonorrhea (22, 6.5%), and syphilis (19, 5.6%). Using binary logistic regression, the predictor of bacterial STI was female gender (odds ratio (OR): 2.033, 95% CI: 1.288-3.208), and knowledge about STI was protective (OR: .640, 95% CI: 0.403-1.014). On the contrary, for viral STIs (including HIV), female gender (OR: .120, 95% CI: .060-.241) and late age of sexual debut (OR: 0.891, 95% CI: .779-1.019) were protective. The predictors of fungal STIs were older age (OR: 1.077, 95% CI: 1.027-1.130), and correct and consistent use of condoms was a protective factor (OR: .279, 95% CI: 0.083-0.943).

Conclusion: The study concluded that female participants had significantly younger ages of sexual debut, and they also had higher chances of bacterial STIs. Genital syphilis and herpes were the most common ulcerative STIs, whereas BV and trichomoniasis were the most common causes of genital discharge.

## Introduction

Sexually transmitted infections (STIs) are diseases that are transmitted by sexual intercourse, which requires the agent to be present in one partner and the other partner to be susceptible to that agent. Apart from these agents, there are host and environmental factors that are needed for STIs to develop. The STIs differ from sexually transmitted diseases (STDs) in that STDs conventionally include clinical features involving the genitalia and other parts of the body, whereas STIs also include infections that may be asymptomatic, e.g., hepatitis B, human T-cell lymphotropic virus type 1 (HTLV-I), etc. [1]. Thus, human immunodeficiency virus (HIV) infection is an STI, whereas acquired immune deficiency syndrome (AIDS) is an STD. The World Health Organization (WHO) has estimated that globally approximately 340 million new cases of the four main curable STIs, i.e., gonorrhea, chlamydia, syphilis, and trichomoniasis, occur every year [2]. Around 75% to 85% of these 340 million cases occur in low- and middle-income countries (LMICs), including India [3]. India accounts for about 30 million STI cases, and it leads to around 17% of productive years lost [4].

Sexually transmitted infections have a tremendous impact on the health burden in India. They are responsible for a significant portion of maternal mortality, ectopic pregnancy, infant illness and death, malignancies, infertility, and increased susceptibility to HIV infection. A variety of demographic and medical factors contribute to the high prevalence of STIs, especially in India, where 50% of the population is in the reproductive age group of 15 to 45 years [5]. Rural-to-urban migration has led to family separation and unbalanced sex ratios in both rural and urban areas, loss of traditional values of sexual behavior, and increased sexual promiscuity. The stigma associated with STIs and embarrassment may prevent infected individuals from seeking medical treatment, thereby increasing the reservoir of infections. These demographic factors have been well documented in understanding the epidemiology and social determinants of STIs, including HIV in India [6]. These factors are age, gender, race, marital status, socio-economic status, education, awareness, etc.

Previous studies on STIs have shown that epidemiology has changed from the 1970s to 2000 in various regions of our country. There has been a reduction in the frequency of donovanosis, an STI that was commonly seen in the 1970s and 1980s [7]. Simultaneously, there was an increase in genital herpes and syphilis cases, and there have been reports of increased HIV seropositivity in these STI cases [8]. It is well known now that the presence of ulcerative STIs can increase the transmission of HIV [9].

The National AIDS Control Organization (NACO) of India has identified surveillance and prompt treatment of STIs as important thrust areas for HIV prevention in the country [10]. The spread of HIV since the late 1980s, with subsequent behavioral change, has resulted in significant alterations in STI epidemic patterns, with a relative fall in the incidence of traditional infections like chancroid, lymphogranuloma venereum (LGV), and donovanosis and a significant rise in viral STIs [11].

With the introduction of the syndromic approach in 1991 by the WHO, which was implemented in India in 2000, knowledge about the relative prevalence of different STIs in the region must help the health authorities make guidelines for their treatment and monitoring [12]. The lack of evidence on this aspect from various parts of India has made it imperative to generate the required evidence on the prevalence and morbidity pattern of common STIs. The current study was conducted to determine the morbidity pattern of different STIs and their determinants at Burdwan Medical College, a tertiary teaching hospital in Burdwan, Eastern India.

## Materials and methods

This descriptive cross-sectional study was conducted in the STI clinic of the Department of Dermatology, Venereology, and Leprosy at Burdwan Medical College, a tertiary care hospital in Burdwan, West Bengal, India. The study period was one year from 1^st^ March 2012 to 28^th^ February 2013, and 340 new cases of STIs attending the STI clinic were enrolled for the study. The sample size was calculated by taking the 53% prevalence of RTI/STI among women reported in a previous study, 10% relative precision, 95% CI, and 80% power [13]. The sampling strategy was systematic random sampling, in which every fifth woman attending the clinic was enrolled in the study.

The inclusion criteria were any adult aged 15 years and above who visited the STI clinic outpatient department (OPD) during the study period, irrespective of sexual orientation or pregnancy status. The participants who acquired STIs by other methods than sexual were excluded (e.g., maternal-fetal transmission, blood transfusions, inadvertent needle sticks, sharing needles, or injection equipment with a potentially infected person). A semi-structured questionnaire was used to record the socio-demographic profile, sexual orientation, age at first sexual encounter, number of sexual partners, type of sexual partners, history of sexual abuse, drug abuse, and their knowledge of STIs and safe sex measures. The tool was developed by the investigators themselves using Delphi techniques (Appendix A). 

A dermatology resident doctor elicited a thorough clinical history, a history relevant to genital and extra-genital lesions, and a history of sexual exposure and performed a clinical examination. The inguinal region was inspected and then palpated for evidence of lymphadenopathy. In male patients, the scrotum was inspected for asymmetry and for the presence of any skin lesions, and the penis was inspected from the base to tip, noting any abnormalities. In case of discharge, the origin of the discharge, whether urethral or sub-preputial, was noted, and if no discharge was immediately apparent, the urethra was milked out to note any discharge. The external urethral meatus was inspected for evidence of meatitis, urethral lesions or discharge, and congenital abnormalities. The anal and perianal region was examined in the left lateral or knee-elbow position for the presence of any ulcer, growth, fissure, or discharge.

Female patients were examined in the lithotomy position in the presence of a female assistant. The perineum, vulva, labia majora, and labia minora were examined for any discharge, redness, swelling, excoriation, ulcer, growth, or any other skin lesions. With the help of a bivalved Cusco speculum, the vagina was examined for evidence of vaginitis, and the color, consistency, and odor of any vaginal discharge was noted. The cervical os was examined for any discharge, and bimanual palpation was done to detect any tenderness. In both men and women, the oral cavity was examined for any ulcer, growth, and whether there was a history of oro-genital or oro-anal contact.

Smear from the urethral/cervical/vaginal discharge (UD/CD/VD)/genital ulcer disease (GUD) was stained with Gram’s stain and examined for the presence of polymorphonuclear leukocytes (PMNs); clue cells and the staining characteristics of the organisms were recorded. In suspected cases of bacterial vaginosis (BV), the pH of the vaginal secretion was obtained by a strip of narrow-range pH paper. A whiff test with 10% potassium hydroxide (KOH) was also performed for the cases of suspected BV and diagnosed as per Amsel's criteria. Sub-preputial discharge and VD were dissolved in 10% KOH and examined for *Candida *while hanging drop preparation (wet mount) from VD was prepared to find trichomoniasis. Tissue smears were taken from the genital ulcer edge for the presence of Donovan bodies/elementary and inclusion bodies of LGV, and a Tzanck smear was done to detect multinucleated giant cells in the genital ulcer. Darkfield microscopy was done to detect treponemes, while venereal disease research laboratory (VDRL) testing was done with blood collected from venipuncture. Titers of more than 1:8 were taken as positive for syphilis to avoid errors due to false positivity. A complement fixation test was performed for suspected cases of LGV, and titers greater than 1:64 were considered positive tests. Enzyme-linked immunosorbent assay (ELISA) tests were also done to detect HIV I and HIV II infections.

Informed consent was taken for participation in the study as well as clinical examination, and female participants were examined in the presence of a chaperone. Consent to publish anonymous results was also obtained from the participants. All data were entered in Microsoft Excel spreadsheets (Microsoft Corp., Redmond, WA), and analysis was performed using IBM SPSS Statistics software, version 26.0 (IBM Corp., Armonk, NY). The Strengthening the Reporting of Observational Studies in Epidemiology (STROBE) checklist was used for reporting the findings of this study. The categorical data were tabulated as percentages segregated by gender and presented as pie charts. The association was tested using Chi-square statistics for categorical variables and Student's t-test for continuous variables, and the significance level was kept as p-value < 0.05. Binary logistic regression was performed to find out the predictors of STIs, using socio-demographic variables and sexual history as independent variables and type of STI as the dependent variable. The three assumptions of binary logistic regression, namely the independence of observations, no perfect multicollinearity, and linearity were tested in IBM SPSS Statistics software using a correlation matrix (all values were <0.7), Mahalanobis distance, and linearity checks.

## Results

A total of 340 patients were included in the study between 1^st^ March 2012 and 28^th^ February 2013. Out of a total of 340 participants, 174 (51.2%) were male and 166 (48.8%) were female. There was no statistical difference between male and female participants (p>0.05) (Table 1).

**Table 1:** Sociodemographic profile of the study participants, segregated by gender (N=340) Rs: rupees; HRGs: high-risk groups; LDTs: long-distance truckers; CSWs: commercial sex workers

Categories		Male (%)	Female (%)	Total (%)
Age (in years)	15-19 years	21 (12.1)	11 (6.6)	32 (9.4)
	20-24 years	35 (20.1)	56 (33.7)	91 (26.8)
	25-29 years	56 (32.2)	52 (31.3)	108 (31.8)
	30-34 years	37 (21.3)	33 (19.9)	70 (20.6)
	≥35 years	25 (14.4)	14 (8.4)	39 (11.5)
	Mean (± SD)	27.6 (± 6.4)	26.6 (± 5.6)	
Locality	Urban	128 (73.6)	123 (74.1)	251 (73.8)
	Rural	46 (26.4)	43 (25.9)	89 (26.2)
Education	Illiterate	8 (4.6)	68 (40.9)	76 (22.4)
	Primary	111 (63.8)	85 (51.2)	196 (57.6)
	Secondary and above	55 (31.6)	13 (7.8)	68 (20.0)
Income	< Rs. 5000/month	152 (87.4)	162 (97.6)	314 (92.4)
	Rs. 5000-15000/month	22 (12.6)	4 (2.4)	26 (7.6)
Occupation	Unskilled worker	138 (79.3)	41 (24.7)	179 (52.6)
	Skilled worker	7 (4.0)	0	7 (2.1)
	Student	13 (7.5)	3 (1.8)	16 (4.7)
	Unemployed	11 (6.3)	8 (4.8)	19 (5.6)
	HRG (LDT/CSWs)	5 (2.9)	4 (2.4)	9 (2.6)
	Homemaker	0	110 (66.3)	110 (32.4)
Marital status	Unmarried	66 (37.9)	15 (9.0)	81 (23.8)
	Married	108 (62.1)	151 (90.9)	259 (76.2)
	Total	174	166	340

Among the males, four subjects were men who have sex with men (MSMs), one of whom was a commercial sex worker (CSW). The mean age of the study participants was 27.10 years (± 6.03) with a range of 17 to 45 years. The mean age of males was higher than females, but the difference was not statistically significant (27.6 ± 6.4 versus 26.6 ± 5.6, t=1.5, p=0.127). The maximum number of participants belonged to the age group of 25 to 29 years (108, 31.8%), followed by 20 to 24 years (91, 26.8%) and 30-34 years (70, 20.6%) (Table 1). Three-fourths of the participants were urban residents (251, 73.8%); about equal numbers were literate (264, 77.6%), and the maximum number of participants had a monthly income < rupees (Rs.) 5,000 (314, 92.4%). Among the males, 138 (79.3%) were unskilled workers (including 26 migrant laborers), while two-thirds of the females were housewives (110, 66.3%). A total of nine participants were at high risk of STIs due to their occupation (e.g., long-distance truckers (LDTs) and CSWs).

The mean age at the first sexual encounter was significantly lower in female participants as compared to male participants (18.5 versus 21.2, t=13.85, p=0.000). However, the number of lifetime sexual partners was significantly higher in male participants as compared to female participants (χ²=87.04, p=0.000) (Table 2). Excluding the male CSW who was an outlier, the average number of sexual partners was 3.28. Among the 50 (28.7%) monogamous male participants, 20 (11.5%) had their spouse as their only partner, while among the 131 (78.9%) monogamous female participants, 124 (74.7%) had their spouse as their only partner. Nearly half of the participants (160, 47.1%) had some knowledge about STIs and HIV/AIDS, whereas only 68 (20%) knew about safe sex measures like condom use and avoidance of casual sexual encounters. Only 55 (16.2%) participants gave a history of correct and consistent use of condoms (Table 2).

**Table 2:** Sexual history of the study participants segregated by gender (N=340) STIs: sexually transmitted infections

Sexual history	Male	Female	Total
Age during the first sexual experience (years)	21.2 (± 2.2)	18.5 (± 1.3)	19.9 (± 2.3)
Number of sexual partners			
01	50 (28.7%)	131 (78.9%)	181 (53.2%)
2-5	98 (56.3%)	31 (18.7%)	129 (37.9%)
>5	26 (14.9%)	4 (2.4%)	30 (8.8%)
Awareness of STIs	108 (62.1%)	52 (31.3%)	160 (47.1%)
Awareness about safe sex	47 (27.0%)	21 (12.7%)	68 (20.0%)
Correct and consistent use of condoms	35 (20.1%)	20 (12.0%)	55 (16.2%)
Total	174	166	340

Of the 340 participants, the most common STIs were herpes genitalis (52, 15.3%), an infection caused by *Candida albicans* (49, 14.4%), non-gonococcal urethritis/cervicitis (43, 12.6%), trichomoniasis (42, 12.4%), and genital warts (31, 9.1%). Gonococcal infection (22, 6.5%), syphilis (19, 5.6%), and chancroid (16, 4.7%) were the next common infections, followed by molluscum contagiosum (eight, 2.4%), LGV (six, 1.8%), genital scabies (two, 0.6%), and donovanosis (one, 0.3%). A total of 39 (11.5%) participants had multiple STIs (including HIV and VDRL positivity) at the time of presentation, and 14 (4.1%) were HIV positive. Bacterial vaginosis was reported by 72 (43.4%) female participants (Table 3).

**Table 3:** Morbidity profile of STIs among the study participants segregated by gender (N=340) STI: sexually transmitted infections; HIV: human immunodeficiency virus

Diagnosis of STI	Male (N=174, %)	Female (N=166, %)	Total (N=340, %)
Herpes genitalis	47 (27.0)	5 (3.0)	52 (15.3)
Genital candidiasis	26 (53.1)	23 (13.9)	49 (14.4)
Non-gonococcal urethritis/ cervicitis	30 (17.2)	13 (7.8)	43 (12.6)
Trichomoniasis	5 (2.9)	37 (22.3)	42 (12.4)
Genital wart	25 (14.4)	6 (3.6)	31 (9.1)
Gonococcal infections	14 (8.0)	8 (4.8)	22 (6.5)
Syphilis	11 (6.3)	8 (4.8)	19 (5.6)
Chancroid	12 (6.9)	4 (2.4)	16 (4.7)
HIV	9 (5.2)	5 (3.0)	14 (4.1)
Molluscum contagiosum	2 (1.1)	6 (3.6)	8 (2.4)
Lymphogranuloma venereum	5 (2.9)	1 (0.6)	6 (1.8)
Genital scabies	2 (1.1)	0	2 (0.6)
Donovanosis	1 (0.6)	0	1 (0.3)

**Figure 1:**
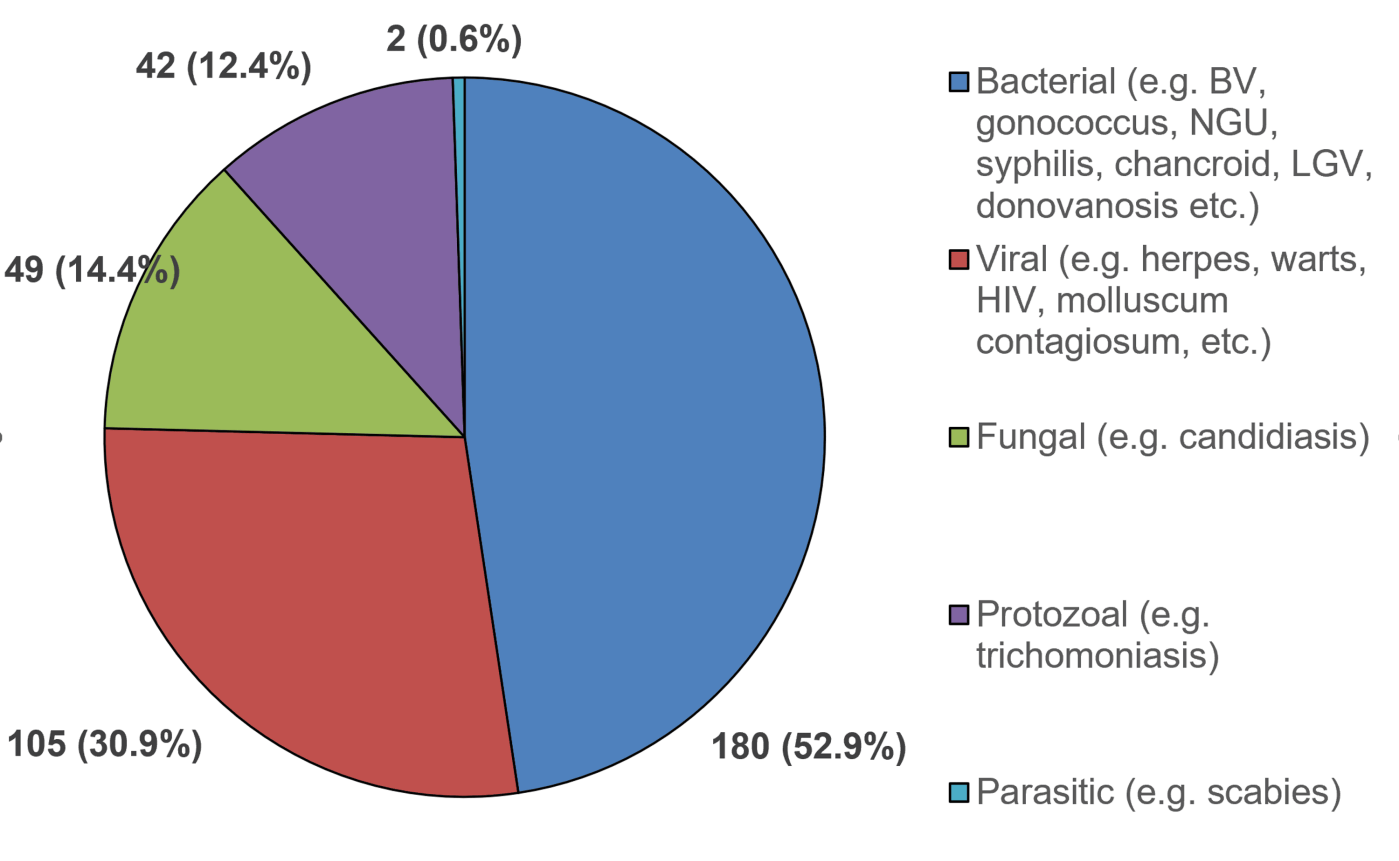
Causative organisms of the STIs diagnosed among the study participants (N=340) STI: Sexually transmitted infections; HIV: human immuno-deficiency virus; BV: bacterial vaginosis; NGU: non-gonococcal urethritis/cervicitis; LGV: lymphogranuloma venereum

Overall, genital discharge (228, 67.0%) was the most common presentation, followed by GUD (94, 27.6%). Effectively, the causative organisms in descending order were bacterial (180, 52.9%), viral (105, 30.9%), fungal (49, 14.4%), protozoal (12.4%), and parasitic (2, 0.6%) (Figure 1).

Among the male homosexual participants (n=4), one was married and the rest were unmarried. One of them was a CSW (transvestite). Three of them had receptive anal intercourse, and all of them reported oro-genital contact. Among the four, the first had perianal and oral warts; the second had a genital wart and gonococcal proctitis; the third had only a genital wart; and the fourth (CSW) had donovanosis. Among the HIV-positive subjects, nine were male and five were female. Out of the 14 HIV-positive people, three were unmarried, and the rest of them were married. Among the male HIV-positive participants, six were unskilled workers, two were migrant laborers, and one was LDT. All of them had contact with multiple CSWs during their lifetime. One participant presented with a history of repeated exposure to CSWs, weight loss, and fever. On screening, he was positive for HIV. Among the rest, five presented with genital herpes, two with NGU, and one with genital candidiasis.

Among the HIV-positive female subjects, one was a CSW who presented with BV and trichomoniasis. The remaining three were housewives, who presented with genital warts, chancroid, and genital candidiasis. All three had their spouse as the only sexual partners, who happened to be migrant laborers.

Univariate analysis was done to find out the predictors of STIs, using socio-demographic variables and safe sex knowledge as independent variables and type of STI as the dependent variable. The association of bacterial STIs was significant with female gender and poor knowledge about STDs. On the contrary, the association of viral STIs (including HIV) was significant with male gender, less education, being married, and incorrect use of condoms. The association of fungal STIs was significant with middle age, lower education, and incorrect use of condoms (Table 4).

**Table 4:** Univariate analysis showing the type of STIs versus socio-demographic variables of the study participants (N=340) *statistically significant; χ2: chi-square value; p-value: probability; STIs: sexually transmitted infections

Categories		Bacterial STIs			Viral STIs			Fungal STIs			
Age (in years)		Yes (%)	No (%)	χ2, p-value	Yes (%)	No (%)	χ2, p- value	Yes (%)	No (%)	χ2, p- value	Total (%)
	15-19 years	15 (9.2)	17 (9.6)	2.85, 0.582	11 (12.2)	21 (8.4)	1.98, 0.739	3 (6.0)	29 (10.0)	10.44, 0.007*	32 (9.4)
	20-24 years	50 (30.7)	41 (23.2)		23 (25.6)	68 (27.2)		9 (18.0)	82 (28.3)		91 (26.8)
	25-29 years	49 (30.1)	59 (33.3)		28 (31.1)	80 (32.0)		16 (32.0)	92 (31.7)		108 (31.8)
	30-34 years	33 (20.2)	37 (20.9)		20 (22.2)	50 (20.0)		10 (20.0)	60 (20.7)		70 (20.6)
	≥35 years	16 (9.8)	23 (13.0)		8 (8.9)	31 (12.4)		12 (24.0)	27 (9.3)		39 (11.5)
Gender	Male	66 (40.5)	108 (61.0)	14.31, 0.00*	73 (81.1)	101 (40.4)	43.89, 0.000*	26 (52.0)	148 (51.0)	0.016, 1.00	174 (51.2)
	Female	97 (59.5)	69 (39.0)		17 (18.9)	149 (59.6)		24 (48.0)	142 (49.0)		166 (48.8)
Locality	Urban	127 (77.9)	124 (70.1)	2.71, 0.109	63 (70.0)	188 (75.2)	0.93, 0.332	34 (68.0)	217 (74.8)	1.03, 0.302	251 (73.8)
	Rural	36 (22.1)	53 (29.9)		27 (30.0)	62 (24.8)		16 (32.0)	73 (25.2)		89 (26.2)
Education	Illiterate	37 (22.7)	39 (22.0)	4.43, 0.109	6 (6.7)	70 (28.0)	19.29, 0.000*	17 (34.0)	59 (20.3)	4.94, 0.038*	76 (22.4)
	Primary	101 (62.0)	95 (53.7)		58 (64.4)	138 (55.2)		26 (52.0)	170 (58.6)		196 (57.6)
	Secondary and above	25 (15.3)	43 (24.3)		26 (28.9)	42 (16.8)		7 (14.0)	61 (21.0)		68 (20.0)
Marital status	Unmarried	33 (20.2)	48 (27.1)	2.21, 0.161	34 (37.8)	47 (18.8)	13.13, 0.000*	8 (16.0)	73 (25.2)	1.977, 0.208	81 (23.8)
	Married	130 (79.8)	129 (72.9)		56 (62.2)	203 (81.2)		42 (84.0)	217 (74.8)		259 (76.2)
Knowledge about STIs	Yes	64 (39.3)	96 (54.2)	7.64, 0.007*	54 (60.0)	106 (42.4)	8.22, 0.005*	22 (44.0)	138 (47.6)	0.22, 0.649	160 (47.1)
	No	99 (60.7)	81 (45.8)		36 (40.0)	144 (57.6)		28 (56.0)	152 (52.4)		180 (52.9)
Knowledge about safe sex	Yes	32 (19.6)	37 (20.9)	0.085, 0.789	24 (26.7)	45 (18.0)	3.07, 0.093	7 (14.0)	62 (21.4)	1.44, 0.260	69 (20.3)
	No	131 (80.4)	140 (79.1)		66 (73.3)	205 (82.0)		43 (86.0)	228 (78.6)		271 (79.7)
Correct and consistent use of condoms	Yes	23 (14.1)	32 (18.1)	0.986, 0.377	27 (30.0)	28 (11.2)	17.25, 0.000*	3 (6.0)	52 (17.9)	4.48, 0.037*	55 (16.2)
	No	140 (85.9)	145 (81.9)		63 (70.0)	222 (88.8)		47 (94.0)	238 (82.1)		285 (83.8)
	Total	163	177		90	250		50	290		340

Binary logistic regression was done taking into account those variables that had univariate p-values < 0.1 in association tables. Sociodemographic variables, sexual practices, and knowledge were taken as independent variables, while type of STI was the dependent variable. For bacterial, viral, and fungal STIs, the final, most parsimonious model had Cox and Snell R² values of 5.9%, 16.5%, and 11.5%, respectively, and Nagelkerke R² values of 7.9%, 24.1%, and 21.0%, respectively. The predictor of bacterial STI was female gender (odds ratio (OR): 2.033, 95% CI: 1.288-3.208), and knowledge about STI was protective (OR: .640, 95% CI: 0.403-1.014). On the contrary, the predictor for viral STIs (including HIV) was female gender (OR: .120, 95% CI: .060-.241) and late age of sexual debut (OR: 0.891, 95% CI: .779-1.019) as protective factors. The predictors of fungal STIs were older age (OR: 1.077, 95% CI: 1.027-1.130), and correct and consistent use of condoms was a protective factor (OR: .279, 95% CI: 0.083-0.943) (Table 5).

**Table 5:** Binary logistic regression for finding predictors of types of STI infections (N=340) STI: sexually transmitted infections; df: degrees of freedom; p-value: probability

Bacterial STI	Beta coefficient	Wald	df	Odds ratio (95% CI)	p-value
Female gender	.709	9.275	1	2.033 (1.288-3.208)	.002
Rural locality	-0.477	3.392	1	0.620 (0.373-1.031)	0.065
Knowledge about STIs	-0.477	3.606	1	0.640 (0.403-1.014)	0.058
Viral STI					
Age of sexual debut	-0.115	2.827	1	0.891 (0.779-1.019)	0.093
Female gender	-1.811	35.625	1	0.120 (0.060-0.241)	.000
Condom use	1.144	11.926	1	3.139 (1.64-6.01)	.001
Fungal STI					
Current age	.074	9.178	1	1.077 (1.027-1.130)	.002
Condom use	-1.276	4.220	1	0.249 (0.083-0.943)	.040

## Discussion

There is a dearth of evidence regarding the epidemiology of STIs in India in general and Eastern and North-Eastern India in particular. The reasons cited are lack of interdepartmental coordination for research, poor attendance of STI patients at public clinics and academic institutions, and availability of limited diagnostic facilities [14]. This study offers important insights into the burden of various STIs and the associated demographic parameters from a tertiary teaching hospital in Burdwan, Eastern India.

In the current study, the proportion of male participants to female participants was 1.05, while previous studies from India reported a higher male-to-female ratio ranging from 1.3:1 to 11:1 [15,16]. Previous studies have stated that reasons for the lower number of female patients seeking treatment in STI clinics are related to the complex interplay of stigma, shyness, poor health-seeking behavior, and preference for female doctors, especially gynecologists [17,18]. Other reasons are the asymptomatic nature of infections in females, a lesser degree of freedom for women to go outdoors, and lower awareness amongst women of the need for medical facilities [19]. In the current study, a higher proportion of female participants was reported, which can be attributed to good inter-departmental coordination between gynecology and dermatology departments for prompt referral, as well as social factors related to female empowerment.

The majority of the clinic attendees in the current study were young urban residents aged between 20 and 29 years. This is quite intuitive, as favorable attitudes towards premarital and extramarital sex, a recreational view of sexuality and sexual adventuring, and the presence of a migrant population who have relatively less social inhibition towards promiscuity might be the reasons behind this. While illiteracy or poverty itself is not a direct risk factor for STI [20], it may lead to population vulnerability for STI [21], as evidenced by the higher proportions of lesser educated and poor STI patients in the current study. Another factor influencing this pattern is that most of these studies are conducted in institutions, whose facilities are availed mainly by economically weaker sections of society.

In the current study, the average age of sexual debut was 20.54 years (21.20 years in male participants and 18.51 years in female participants). Many previous studies have reported an early age of sexual debut in female subjects as compared to male subjects [22]. In one South Indian study, the average age of sexual debut in STI clinic attendees was 20.01 ± 3.78 years [23]. Early sexual debut leads to higher rates of partner exchange and greater chances of STI transmission [24].

In the current study, the most common category of STI was genital discharge, including *Candida *infection (49, 14.4%), trichomoniasis (42, 12.4%), NGU (43, 12.6%), and gonococcal infection (22, 6.5%). Bacterial vaginosis is not considered an STI per se, with incidence varying according to the population studied and geographic location (2.5% to 48%) [25]. Most of the BV cases in the current study had their spouse as the only sexual partner (88%). This could be explained by the fact that BV is not just correlated with the number of lifetime sexual partners but also influenced by other factors like increasing years since marriage, lower socioeconomic status, parity more than two, stage of menstrual cycle, and hours since last intercourse. Similarly, the incidence rate of NGU reported in the literature varies from 4% to 15% among heterosexual males attending STI clinics [26]. In the current study, 6.5% of subjects presented with gonococcal infection, and a previous study from Thailand had shown that 6.0% (95% CI: 5.1% to 6.9%) of the male subjects with a history of GUD presented with gonorrhea with an incidence rate of 1.3 (95% CI: 1.1-1.5) per 100 person-years [27].

The second most common category was GUD, including herpes genitalis (52, 15.3%), syphilis (19, 5.6%), chancroid (16, 4.7%), and LGV (6, 1.7%). Herpes is the leading cause of GUD globally, followed by *Treponema pallidum* (syphilis), *Haemophilus ducreyi* (chancroid), and *Chlamydia trachomatis* serovars L1-L3 (LGV) [28].

The changing trend of STIs in Northern India shows that between the 1990s and 2004, there has been a gradual decline in cases of some STIs, like donovanosis, with a similar increase in other GUDs, like herpes and syphilis [29]. A similar pattern has also been seen in Eastern India [16], and the current study also shows a similar pattern with very few donovanosis cases and a high number of syphilis cases. However, the proportion of genital discharge diseases was disproportionately high. The likely reason might be that BV was included as a genital discharge STI in the current study.

In our study, genital syphilis (15.29%) and herpes (5.58%) were the two most common ulcerative STIs, and BV (21.17%) was the most common genital discharge disease. As per the annual report of the National AIDS & STD Response, the seropositivity of syphilis detected by the rapid plasma regain test among STI clinic attendees in West Bengal was 1.61% in 2023 [30]. The Global Burden of Disease study in 2019 also concluded that over the past 20 years, the age-standardized incidence rates have remained stable for trichomoniasis and genital herpes, decreased for chlamydia and gonorrhea, and increased for syphilis [31].

The seropositivity of HIV among the subjects was 4.12%. The annual report of the National AIDS & STD Response in 2023 reported a 0.08% prevalence of HIV cases among adults (15-49 years) in West Bengal [30]. The proportions of STI clinic attendees with syphilis and HIV in the current study were obviously higher than the general population prevalence reported by NACO, as it was a hospital-based study.

The strength of the current study lies in the fact that it tried to find an association of types of STIs with various sociodemographic and sexual history variables. It found higher bacterial STIs in females, higher viral STIs in males, and higher fungal STIs among the elderly. Also, correct and consistent use of condoms and good knowledge about STIs emerged as important protective factors. The study had few limitations. Firstly, a hospital-based study suffers from poor generalizability, as external validity is limited by the purposive nature of sampling. Secondly, a cross-sectional study cannot elucidate causal pathways as to the etiology of STIs. A smaller sample size also precludes the estimation of the actual prevalence of STIs in the community or any approximation of the predictors of diseases over time. Further studies with a larger sample size and adequate power to elucidate predictors might provide greater insight into determinants of STIs in India.

## Conclusions

The epidemiology of STIs results from the interaction between STI pathogens, the behaviors that help transmit them, and the effectiveness of prevention and control interventions. The study concluded that STIs are equally common among both male and female subjects, but they tend to cluster in sexually active and economically productive age groups, especially female subjects aged between 20 to 30 years. The predictor of bacterial STIs was lack of awareness, while female gender and number of sexual partners were predictors of viral STIs. The predictors of fungal STIs were female gender and older age. There is a strong association between the acquisition of STIs and a young age at first intercourse. Genital discharge was the most common presentation among the STI clinic attendees, followed by GUD.

## Appendices

Appendix A

Performa for Recording the Data

Name:                                                Age/Sex                                    Date:

Residence:                                         Qualification:                          Religion:

Annual income:                         Profession: _________                     /Student/housewife/sex worker/migrant labor.

Married/unmarried/single/widow/divorcee

Sexual orientation: natural/un-natural (specify)______________.

Currently active sexually: yes/no

Age at first sex:

Number of sexual partners:  lifetime:         during the last one year:

Type of partners: casual/steady CSW/spouse/girlfriend/boyfriend/other

History of sexual abuse:  yes/no, details if yes.

Use of drug abuse: IDR/others.

Knowledge about sex and sexually transmitted diseases: yes/no

Consciousness about safe sex measures: yes/no

Do you know that the use of condoms prevents STIs including HIV: yes/no

Have you ever used a condom: yes/no

Do you practice safe sex: yes/no/always/often

Are you willing to practice safe sex in the future: yes/no/always/often

Clinical records:

Presenting complaints:

Genital ulcer/growth/features of urethritis/genital discharge/others (specify)

Duration:

History of exposure: yes/no

Last exposure:                                         time between exposure and symptoms:            

 Frequency of exposure if repeated exposure:

Any history of un-natural sexual behavior ( specify)

Past history of STDs: yes/no.

Details, if any:

Have you taken any treatment for the present complaint: yes/no(specify)

Any other medical history:

Examination:

Local(genital) examination:

Lymph node enlargement: yes/no. Group:

Systemic examinations:

Investigations:

Gram staining:

Dark ground microscopy: positive/negative

KOH mount: positive/negative

Hanging drop preparation: positive/negative.

Tzank smear: positive/negative

VDRL: positive/negative            titer:

HIV: positive/negative

Others :

Treatment given:

Counseling:

Follow up:

1st:

2nd:
